# Antioxidant and Antiplasmodial Activities of Bergenin and 11-*O*-Galloylbergenin Isolated from *Mallotus philippensis*

**DOI:** 10.1155/2016/1051925

**Published:** 2016-02-22

**Authors:** Hamayun Khan, Hazrat Amin, Asad Ullah, Sumbal Saba, Jamal Rafique, Khalid Khan, Nasir Ahmad, Syed Lal Badshah

**Affiliations:** ^1^Department of Chemistry, Islamia College University, Peshawar 25120, Pakistan; ^2^Institute of Chemical Sciences, University of Peshawar, Peshawar 25120, Pakistan; ^3^Departamento de Química, Universidade Federal de Santa Catarina, 88040-900 Florianopolis, SC, Brazil

## Abstract

Two important biologically active compounds were isolated from* Mallotus philippensis*. The isolated compounds were characterized using spectroanalytical techniques and found to be bergenin (**1**) and 11*-O*-galloylbergenin (**2**). The* in vitro* antioxidant and antiplasmodial activities of the isolated compounds were determined. For the antioxidant potential, three standard analytical protocols, namely, DPPH radical scavenging activity (RSA), reducing power assay (RPA), and total antioxidant capacity (TAC) assay, were adopted. The results showed that compound** 2** was found to be more potent antioxidant as compared to** 1**. Fascinatingly, compound** 2 **displayed better EC_50_ results as compared to *α*-tocopherol while being comparable with ascorbic acid. The antiplasmodial assay data showed that both the compound exhibited good activity against chloroquine sensitive strain of* Plasmodium falciparum *(D10) and IC_50_ values were found to be less than 8 *μ*M. The* in silico* molecular docking analyses were also performed for the determination of binding affinity of the isolated compounds using* P. falciparum* proteins PfLDH and Pfg27. The results showed that compound** 2** has high docking score and binding affinity to both protein receptors as compared to compound** 1**. The demonstrated biological potentials declared that compound** 2** could be the better natural antioxidant and antiplasmodial candidate.

## 1. Introduction

Medicinal plants, their extracts, or the isolated purified constituents have been used extensively as remedy for treatment of several diseases. The flora of Pakistan is quite rich with the naturally gifted medicinal plants. However, very little attention was given to explore the medicinal potentials of such worthy materia medica [1–3]. Various ailments such as Parkinson's, Alzheimer's, cancer, inflammation, neurodegeneration, aging, injury to blood vessel membranes, heart, and brain, and a number of other diseases may be caused by the free radicals present in the body. Antioxidants are free radical scavengers that may prevent, protect, or reduce the extension of such damage [4, 5]. A number of chemical species including both synthetic and natural products may act as antioxidants. Plants are considered to be the best source of natural antioxidants [1–3, 6]. Similarly, the various antimalarial and antiparasitic drugs such as quinine and artemisinin were also reported from the medicinal plants [7].

Bergenin and 11*-O*-galloylbergenin are the two natural products. The biological and pharmacological activities of bergenin are well documented in the literature [8–21]. However, very little work has been reported on the biological potentials of 11*-O*-galloylbergenin [22, 23] which need to be more explored. In the present study, the abovementioned natural products were isolated from* Mallotus philippensis* and their antioxidant and antiplasmodial activities were investigated. For confirmation of the experimental results, the computational study was also performed using* in silico* molecular docking.

## 2. Materials and Methods

### 2.1. General Experimental Procedures

^1^H-NMR and ^13^C-NMR were recorded at 400 MHz for ^1^H and at 100 MHz for ^13^C using TMS as internal standard with Bruker DPX-400 instrument in deuterated solutions. Mass spectra were recorded on Agilent 5973N instrument using EI mode. IR spectra were determined using a Jasco A-302 spectrophotometer. UV and UV-visible spectra were recorded using U-3200 (Hitachi, Japan) and SP-3000 PLUS (Optima, Japan) spectrophotometers. For TLC and column chromatography, aluminum sheets precoated with silica-gel 60 F254 (20 × 20 cm, 0.2 mm thick; E. Merck, Germany) and silica gel (200–300 mesh), respectively, were used. The commercial solvents were used for extraction purpose and were redistilled. For the antioxidant and antiplasmodial activities, analytical grade reagents and chemicals were used.

### 2.2. Plant Material

The stem wood of* M. philippensis* (Euphorbiaceae) was collected from district Bunner located in the north of Pakistan in July 2006 and identified via Voucher Number 1013 (pup) by Professor Dr. Abdur Rashid, Department of Botany, University of Peshawar, Peshawar, Pakistan.

### 2.3. Extraction and Isolation

**Scheme 1:**
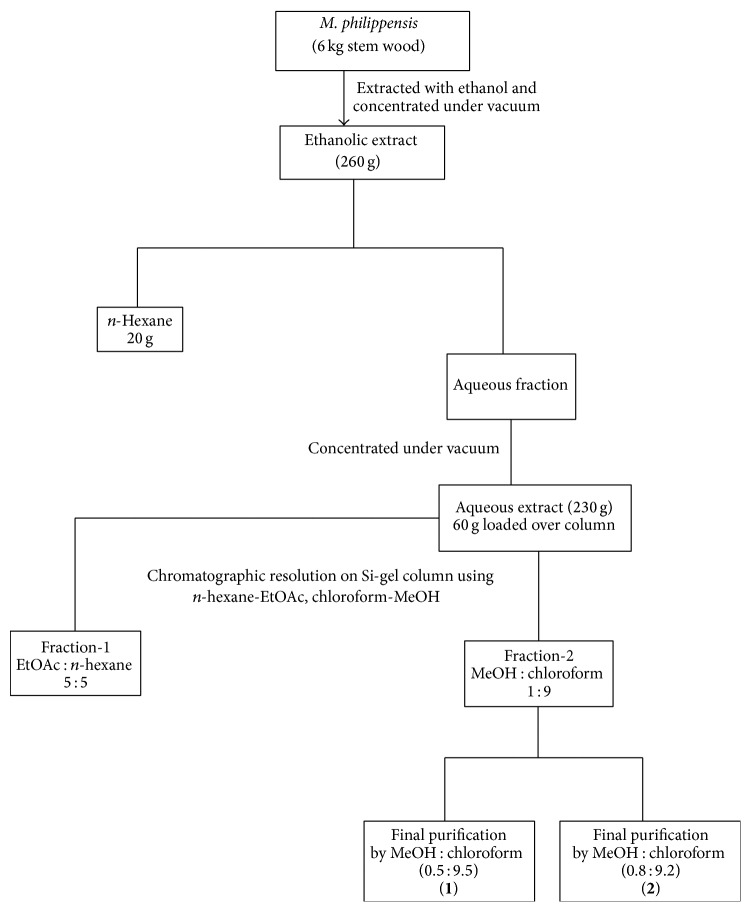
Schematic representation of extraction and isolation of compounds** 1** and** 2** from* Mallotus philippensis* stem wood.

Air dried plant material was chopped, grinded, and extracted three times with commercial ethanol for 72 h which afforded 4.32% of the crude extract. From the crude extract, *n*-hexane soluble fraction was removed by solvent extraction with water. The aqueous fraction was then dried under vacuum which was further processed for the isolation of compounds** 1 **and** 2 **as depicted in Scheme 1. The isolated compounds were characterized using various spectroanalytical techniques.

### 2.4. Antioxidant Potential

The antioxidant potential of the isolated compounds was determined using DPPH radical scavenging activity (RSA) [1–3, 24], reducing power assay (RPA) [24, 25] and total antioxidant capacity (TAC) assay [26].

### 2.5. Antiplasmodial Activity

For antiplasmodial activity, the isolated compounds were tested against chloroquine sensitive (CQS) strain of* Plasmodium falciparum *(D10). Continuous* in vitro* cultures of asexual erythrocyte stages of* P. falciparum* were maintained using the reported method [27]. For the quantitative assessment of antiplasmodial activity, parasite lactate dehydrogenase assay was adopted [28]. The IC_50_ values were obtained using a nonlinear dose-response curve fitting analysis via Graph Pad Prism v.4.0 software.

### 2.6. Molecular Docking

For the* in silico* molecular docking study, the crystal structures of receptor proteins were downloaded from the protein data bank, code number PfLDH (*P. falciparum* lactate dehydrogenase) and PFG27 (gametocyte protein) of* P. falciparum*. The water molecules were removed and 3D protonation of the receptor molecules was carried out. The energies of the retrieved receptors were minimized using the default parameters of MOE energy minimization algorithm (gradient: 0.05, force field: MMFF94X). For the molecular docking of the isolated compounds, default parameters of MOE-dock program were used. To find the correct conformations of the ligands and to obtain minimum energy structure, ligands were allowed to be flexible. At the end of docking, the best conformations of the ligand were analyzed for their binding interactions.

## 3. Results and Discussion

### 3.1. Characterization of the Isolated Compounds

The spectral analyses of the two isolated compounds are summarized as follows.

*Compound* ***1***. White needles; mp = 237°C; UV *λ*_max_ (log *ε*) = 279(4.28); IR (KBr) *λ*_max_ = 3310, 3350, 1712, 1632, 1609, 1510, 1230 cm^−1^; ^1^H-NMR (DMSO-d_6_, 400 MHz): *δ* 6.97 (1H, s, H-7), 5.67 (1H, d, H-10b), 4.98 (1H, dd, H-4a), 3.89 (1H, dd, H-4), 3.81 (2H, d, H-11), 3.75 (3H, s, H-12), 3.62 (1H, m, H-2), 3.48 (1H, dd, H-3); ^13^C-NMR (DMSO- d_6_, 100 MHz): *δ* 60.0 (C-12), 61.1 (C-11), 70.7 (C-3), 72.1 (C-10b), 73.7 (C-4), 79.8 (C-4a), 81.7 (C-2), 109.4 (C-7), 116.0 (C-10a), 118.1 (C-6a), 140.6 (C-9), 148.1 (C-10), 151.0 (C-8), 163.5 (C-6); EIMS* m/z* (rel. int.): 328 (34), 208 (100), 237 (7), 170 (30).

**Figure 1:**
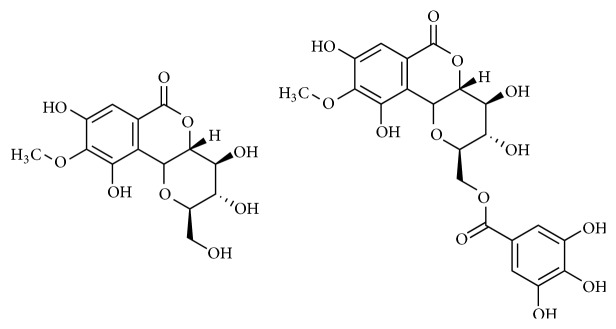
Chemical structure of isolated compounds** 1** (bergenin) and** 2** (11*-O*-galloylbergenin).

Compound** 1** was obtained as white crystals. The mass spectral data of compound** 1** gave a molecular ion peak at* m/z* 328 which leads to a molecular formula of C_14_ H_16_O_9_. The melting point of 237°C is consistent with the published melting point of bergenin, that is, 238°C. The ^1^H-NMR spectral analysis showed a signal for one aromatic proton at *δ* 6.97 (1H, s) and a signal for methoxy protons at *δ* 3.8 (3H, s). In the ^13^C-NMR spectrum, the signals were observed at *δ* 163.5 and 60.0 for carbonyl and methoxy groups, respectively. Compound** 1 **was characterized as bergenin (Figure 1) by comparing its physical and spectral data with previous literature [23, 29].

*Compound* ***2***. White amorphous powder; mp = 180°C; [*α*]_D_^15^ = +37.6° (EtOH; c 1.2), UV *λ*_max_ (log *ε*) = 277(4.22); IR (KBr) *λ*_max_ = 3310, 1712, 1632, 1609, 1510, 1230 cm^−1^; ^1^H-NMR (90 MHz, Me_2_CO-d): *δ* 7.21(2H, s, gall-H), 7.10 (IH, s, H-7), 3.90 (3H, s, OMe); ^13^C-NMR (90 MHz, CD_3_OD): *δ* 81.2 (C-2), 72.2 (C-3), 75.7 (C-4), 81.1 (C-4a), 166.5(C-6), 120.0, (C-6a), 112.0 (C-7), 153.1 (C-8), 143.3 (C-9), 150.0 (C-10), 117.7 (C-10a), 74.7 (C-10b), 65.1 (C-11), 61.3 (C-12), 121.8 (C-1′), 111.1 (C-2′, C-6′), 147.4 (C-3′, C-5′), 140.0 (C-4′), 169.2 (C-7′); EIMS* m/z* (rel. int.): 480 (32), 328 (34), 208 (100), 237 (7), 170 (30).

Compound** 2** was interpreted and analyzed as C_21_H_20_O_13_ to be monogalloyl ester of bergenin by its ^1^H-NMR, ^13^C-NMR, and EIMS spectral data and also by acid hydrolysis which gave bergenin and gallic acid. The position of the galloyl group was established by ^13^C-NMR spectrum and 2D techniques. The carbon signals other than that of C-11 in the glucose moiety of bergenin were assigned as given below. The carbon carrying free hydroxyl group (C-3, C-4, and C-11) was unequivocally distinguished from the others (C-2, C-4a, and C-10b) by the deuterium induced differential isotope shift (DIS) measurement. Compound** 2 **was characterized as 11-*O*-galloylbergenin (Figure 1) [23, 30].

### 3.2. Antioxidant Activity

In DPPH radical scavenging assay, the isolated compounds** 1** and** 2** showed 6.858 ± 0.329 and 87.26 ± 1.671% RSA, respectively when compared with the selected standards whose %RSA were in the range from 92.26 ± 0.547 to 98.35 ± 0.871 (Table 1). The demonstrated %RSA of compound** 2** clearly indicates that it is the high potency toward DPPH free radical. Similarly, in the RPA, the reducing power capacity of compound** 2** was found to be much higher as compared to compound** 1 **while being comparable with gallic acid and quercetin as depicted in Table 1. The TAC of the isolated compounds and standards was determined as ascorbic acid equivalent as shown in Table 1. As can be seen from the results, again compound** 2** displayed better activity as compared to compound** 1** and even *α*-tocopherol. From the above discussion, compound** 2 **could be declared as the better antioxidant candidate. The antioxidant properties of various plants extract or their purified constituents are well documented in the literature [1–3, 6, 22, 24, 31–34].

Table 2 shows a comparative analysis of EC_50_ values of the isolated compounds and standards using DPPH radical scavenging and reducing power assays. The EC_50_ values showed more prominent performance of compound** 2** as compared to compound** 1**. For the studied assays data, the EC_50_ vales for compound** 2** showed better results as compared to *α*-tocopherol while being comparable with the ascorbic acid (Table 2). In a previous study, the antioxidant activity of various compounds isolated from the methanolic extract of the aerial parts of* Vitex agnus-castus *Linn. plant was evaluated using a DPPH radical scavenging assay and the results obtained were in the range from no activity to strong activity. However, the IC_50_ value was not reported [6]. The results obtained in the present study are comparable with the reported data [22].

### 3.3. Antiplasmodial Activity

Compounds** 1 **and** 2** were also tested for the* in vitro* antiplasmodial activity against the CQS D10 strain of* P. falciparum* and the results obtained are presented in Table 3. As can be seen, both the tested compounds had displayed good activity even at low concentration with IC_50_ values of 6.92 ± 0.43 and 7.85 ± 0.61 *μ*M for compounds** 1 **and** 2**, respectively, while IC_50_ value of 0.031 ± 0.002 *μ*M was recorded for chloroquine (Table 3). The analogous results were also reported previously for mentioned compounds isolated from the roots of* Bergenia ligulata*.

### 3.4. Molecular Docking

The binding interaction of the isolated compounds and* P. falciparum* proteins (PfLDH and Pfg27) was also investigated using* in silico* molecular docking. The selected proteins are very important because PfLDH has a role in glycolysis for energy production during asexual cycle, while Pfg27 is vital protein for the gametocyte production during sexual phase of the parasite; thus both proteinsare potential molecular targets for antimalarial drugs. The results of molecular docking with compound** 1** and PfLDH showed that compound was bound in the binding pocket of the enzyme, making interactions with the residues Lys198, Arg109, Asn108, and Asn197 (basic, side chain donors and backbone donor). Lys98 interacts with oxygen of one hydroxyl group of ring and Arg109 with other hydroxyl group oxygen while oxygen of third hydroxyl group established interaction with the Asn197 whereas the oxygen atom of the ring interacts with Asn108 (Figure S2 in Supplementary Material available online at http://dx.doi.org/10.1155/2016/1051925). Compound** 2** was completely docked in cavity of the enzyme PfLDH and established large number of interactions with the residues Arg185, Ser170, Glu256, Lys173, Val166, Gly165, Thr169, Ala253, and Ala249. In the above docking process, the residue Arg185 formed three interactions, that is, one with oxygen of one hydroxyl group of benzene ring, the second with oxygen of carboxylic group of compound** 2,** and the third (arene-arene interaction) with benzene ring. The residue Ser170 established two interactions (backbone donor and acceptor) with hydrogen of hydroxyl group of two cyclic rings and one with oxygen of hydroxyl group of benzene ring of compound** 2**. The residue Glu256 showed interaction with the hydrogen atom of benzene hydroxyl group (side chain acceptor) and Lys173 has two interactions (side chain donor) with the two oxygen atoms of two hydroxyl groups of benzene ring. The residue Gly165 formed one interaction with hydrogen of hydroxyl group of benzene ring and residue Thr169 (side chain acceptor) showed one interaction with hydrogen of one hydroxyl group. Val166 (backbone acceptor) formed two interactions with the two hydrogen atoms of one hydroxyl group of one benzene ring and with the other hydroxyl group of another benzene ring. Ala253 and Ala249 (backbone donor) both expressed interactions with oxygen of hydroxyl group and carboxyl group (Figure S3).

The results of molecular docking of compound** 1** and Pfg27 protein binding showed that** 1 **was bound into the binding cavity of protein (Pfg27) making interactions with the residues Arg131 (basic, side chain donor) and Asp40. Arg131 interacts with oxygen (carboxyl group) to one side of benzene ring while Asp40 was found in polar interaction with H (hydroxyl group) of compound** 1 **(Figure S4). Similarly, Arg36 residue also showed prominent interaction with oxygen of hydroxyl group as shown in Figure S5. In case of compound** 2**, Arg131 residue established arene-cation interaction with one of the benzene rings of compound and the residue Glu134 formed three-side interaction, that is, two sides with hydrogen of two hydroxyl groups and one side with one oxygen group of carboxyl group (Figure S6). Further, compound** 2** also showed arene-arene interaction the residue His28, while Arg36 and Gln130 formed interaction with hydrogen of hydroxyl groups (Figures S7 and S8). From the MOE-docking studies, it was observed that, for both the proteins, compound** 2** has good agreement of docking score and binding affinity to protein receptors as compared to compound** 1 **as shown in Table 4. The results demonstrated that the isolated compounds are good enough for their potency and effectiveness against* P.  falciparum*.

## 4. Conclusions

The current study deals with the isolation and characterization of two biologically active compounds from* M. philippensis*. The natural products were found to be bergenin (**1**) and 11*-O*-galloylbergenin (**2**). The isolated constituents were evaluated for their antioxidant and antiplasmodial potentials and from the results it was evident that compound** 2 **was found to be a potent and effective antioxidant as compared to compound** 1** and its synthetic derivatives [8, 30]. The isolated compounds also offered good antiplasmodial activity against the tested* P. falciparum *strain which was further confirmed using* in silico* molecular docking. It is therefore concluded that the demonstrated medicinal properties of the isolated compounds could be used as scaffolds for the generation of advanced natural products and may play a vital role in drug development and design.

## Supplementary Material

In these supplementary materials are related to the *in silico* molecular docking analyses of the isolated compound **1** and **2**. FIGURE S2 to S8 shows 2D ligand interaction diagrams of the compound **1** and **2** (docked ligands) with the active binding sites of *Plasmodium falciparum* (D10) proteins (PfLDH and Pfg27).

## Figures and Tables

**Table 1:** Antioxidant activity of the isolated compounds and standards.

Tested compounds	% radical scavenging activity (RSA)	Reducing power assay (RPA)	Total antioxidant capacity^*∗*^
Bergenin (**1**)	6.858 ± 0.329	0.055 ± 0.002	49.159 ± 3.136
11-*O*-Galloylbergenin (**2**)	87.26 ± 1.671	1.315 ± 0.027	951.50 ± 109.64
Ascorbic acid	97.85 ± 0.623	3.351 ± 0.034	2478.36 ± 173.81
Gallic acid	98.12 ± 0.931	1.435 ± 0.031	2201.05 ± 152.33
Quercetin	98.35 ± 0.871	1.772 ± 0.041	2030.29 ± 134.51
*α*-Tocopherol	92.26 ± 0.547	22.026 ± 0.074	565.17 ± 25.32

Each reading is mean (*n* = 3) ± SD (standard deviation). For RSA and RPA, 100 and 25 *μ*g/mL, respectively, were used. ^*∗*^As ascorbic acid equivalent (*μ*mol/mg).

**Table 2:** EC_50_ values of the isolated compounds and standards.

Tested compounds	Radical scavenging assay (EC_50_) (*μ*g/mL)^a^	Reducing power assay (EC_50_) (*μ*g/mL)^b^
Bergenin (**1**)	99.807 ± 3.120	24.915 ± 1.326
11-*O*-Galloylbergenin (**2**)	7.276 ± 0.058	5.208 ± 0.095
Ascorbic acid	6.571 ± 0.303	3.551 ± 0.073
Gallic acid	4.732 ± 0.187	1.542 ± 0.062
Quercetin	4.355 ± 0.099	2.073 ± 0.065
*α*-Tocopherol	33.675 ± 2.019	22.152 ± 1.153

Each reading is mean (*n* = 3) ± SD (standard deviation). ^a^EC_50_: effective concentration at which 50% of DPPH radicals are scavenged and ^b^EC_50_: effective concentration at which the absorbance is 0.4.

**Table 3:** The *in vitro* antiplasmodial activity of the isolated compounds and standard.

Tested compounds	Antiplasmodial activity (IC_50_ in *μ*M)
Bergenin (**1**)	6.92 ± 0.43
11-*O*-Galloylbergenin (**2**)	7.85 ± 0.61
Chloroquine	0.031 ± 0.002

Each reading is mean (*n* = 3) ± SD (standard deviation).

**Table 4:** The *in silico* docking score of the isolated compounds against *P. falciparum* proteins (PfLDH and Pfg27).

Isolated compounds	Docking result Moldock score			
	PfLDH		PfG27	
	Moldock score	Binding affinity (pKi)	Moldock score	Binding affinity (pKi)
**1**	−12.13	10.20	−10.01	8.78
**2**	−16.22	12.43	−11.84	9.29
