# Supplementary material for: Antioxidant and Antiplasmodial Activities of Bergenin and 11-O-Galloylbergenin Isolated from Mallotus philippensis
Source: Oxid Med Cell Longev. 2016 Feb 22;2016:1051925. doi: 10.1155/2016/1051925 (PMC4779831; doi:10.1155/2016/1051925)
Supplement: Supplementary file 1 — In these supplementary materials are related to the in silico molecular docking analyses of the isolated compound 1 and 2. FIGURE S2 to S8 shows 2D ligand interaction diagrams of the compound 1 and 2 (docked ligands) with the active binding sites of Plasmodium falciparum (D10) proteins (PfLDH and Pfg27). [file 1051925.f1.doc]

**Supporting Information**

**Antioxidant and antiplasmodial activities of bergenin and 11-*O*-galloylbergenin isolated from *Mallotus philippinensis***

**Hamayun Khan,^1^ Hazrat Amin,^2^ Asad Ullah,^1^ Sumbal Saba,^2,3^ Jamal Rafique,^2,3^  Khalid Khan,^1^ Nasir Ahmad,^1^ and Syed Lal Badshah^1^**

^1^ *Department of Chemistry, Islamia College University, Peshawar-25120, Pakistan*

^2^ *Institute of Chemical Sciences, University of Peshawar, Peshawar-25120, Pakistan*

^3^ *Departamento de Química, Universidade Federal de Santa Catarina, Florianopolis*

*88040-900, Brazil*

Correspondence should be addressed to Hamayun Khan; hamayun84@yahoo.com


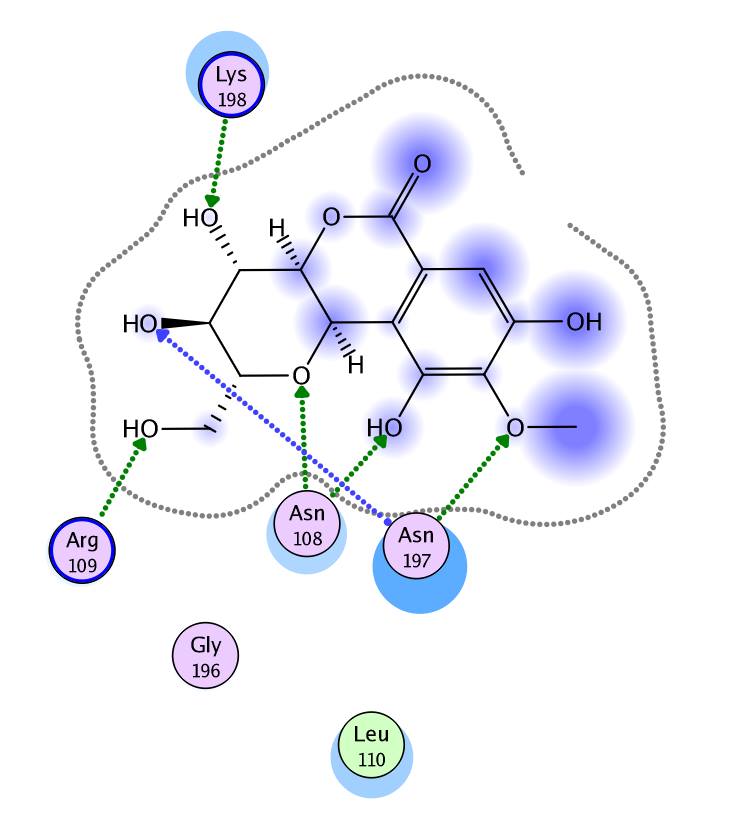


Figure S2: 2D ligand interaction diagram of the docked ligand (compound **1**) within the binding site of the protein of *Plasmodium* PDB code PfLDH. The Figure is rendered by MOE.


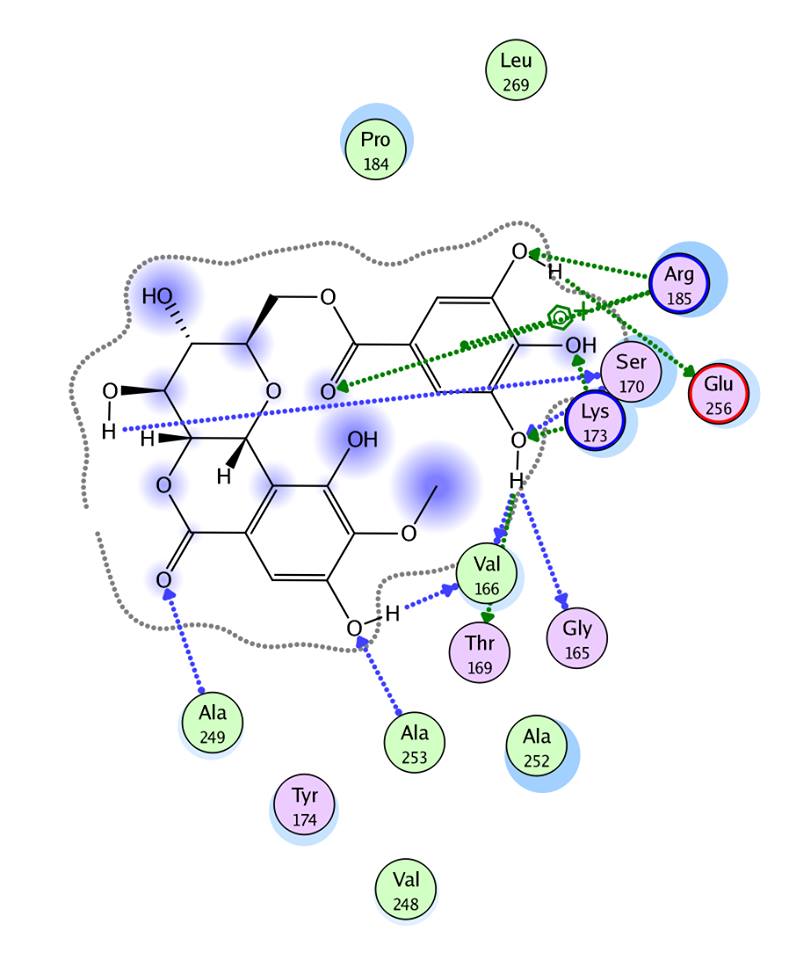


FIGURE S3: 2D ligand interaction diagram of the docked ligand (compound **2**) within the binding site of the protein of *Plasmodium* PDB code PfLDH. Figure is rendered by MOE.

**
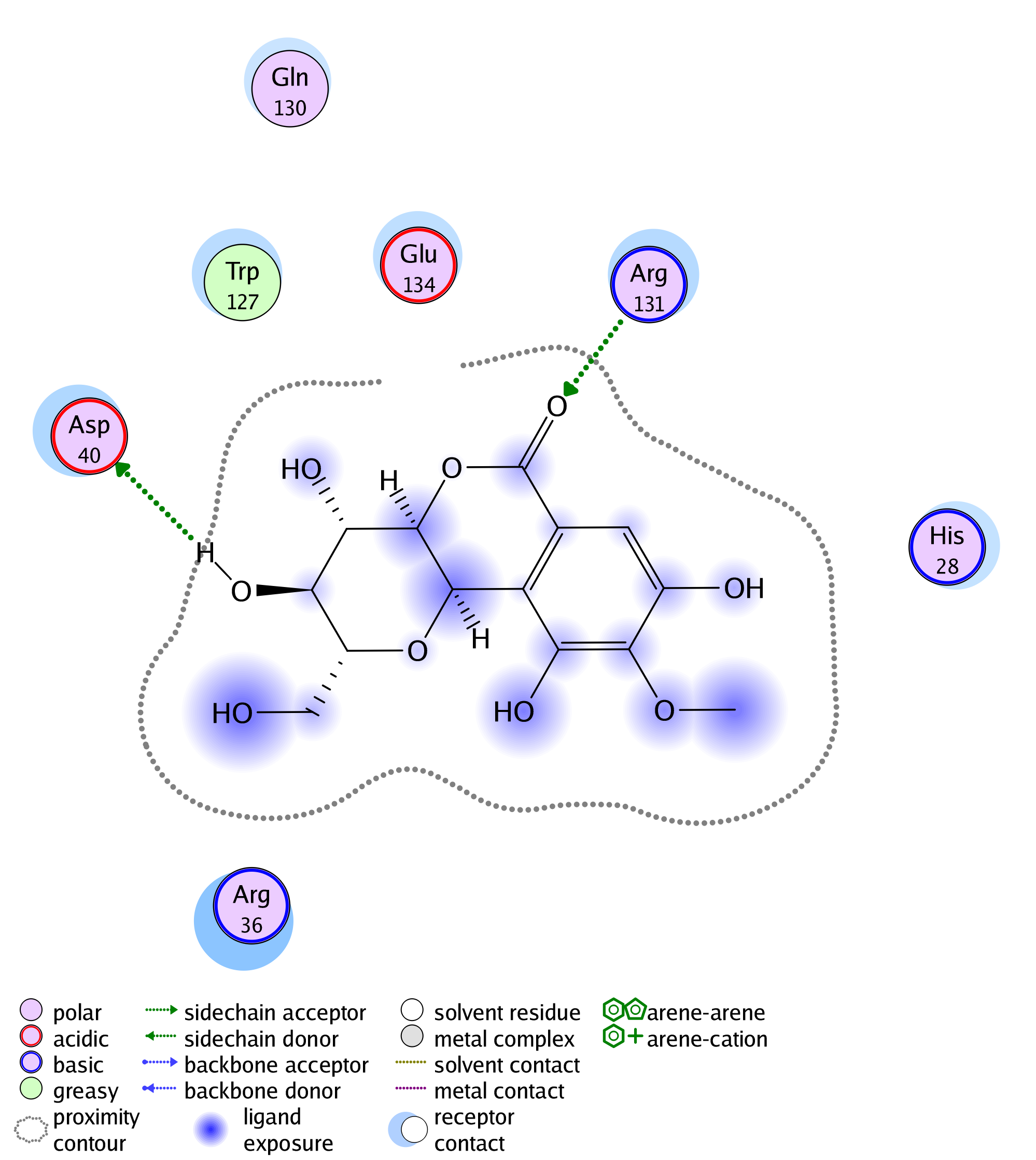
**

FIGURE S4: 2D ligand interaction diagram of the docked ligand (compound **1**) within the binding site of the protein of *Plasmodium* PDB code PFG27.

**
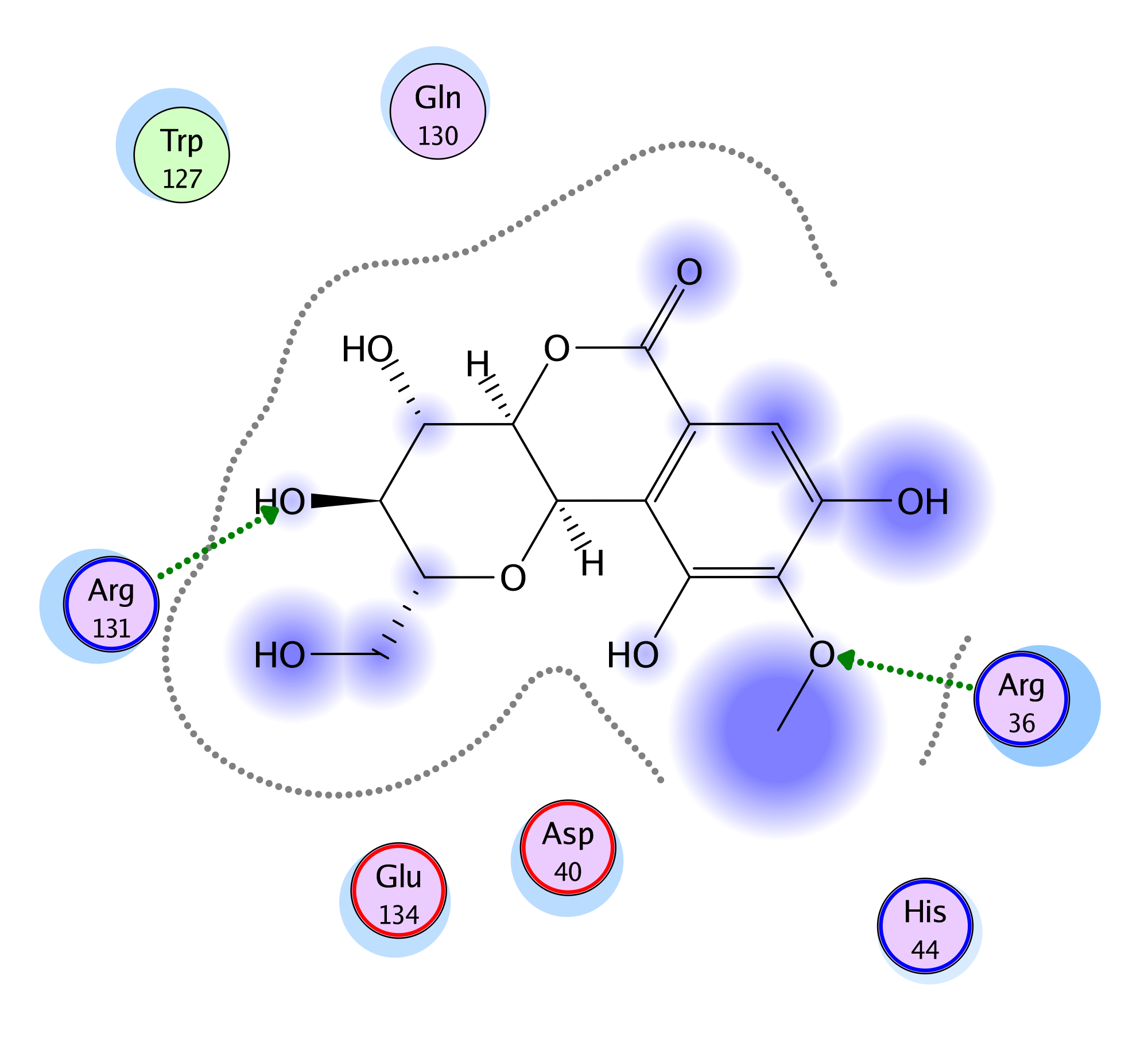
**

FIGURE S5: 2D ligand interaction diagram of the docked ligand (compound **1**) within the binding site of the protein of *Plasmodium* PDB code PFG27.


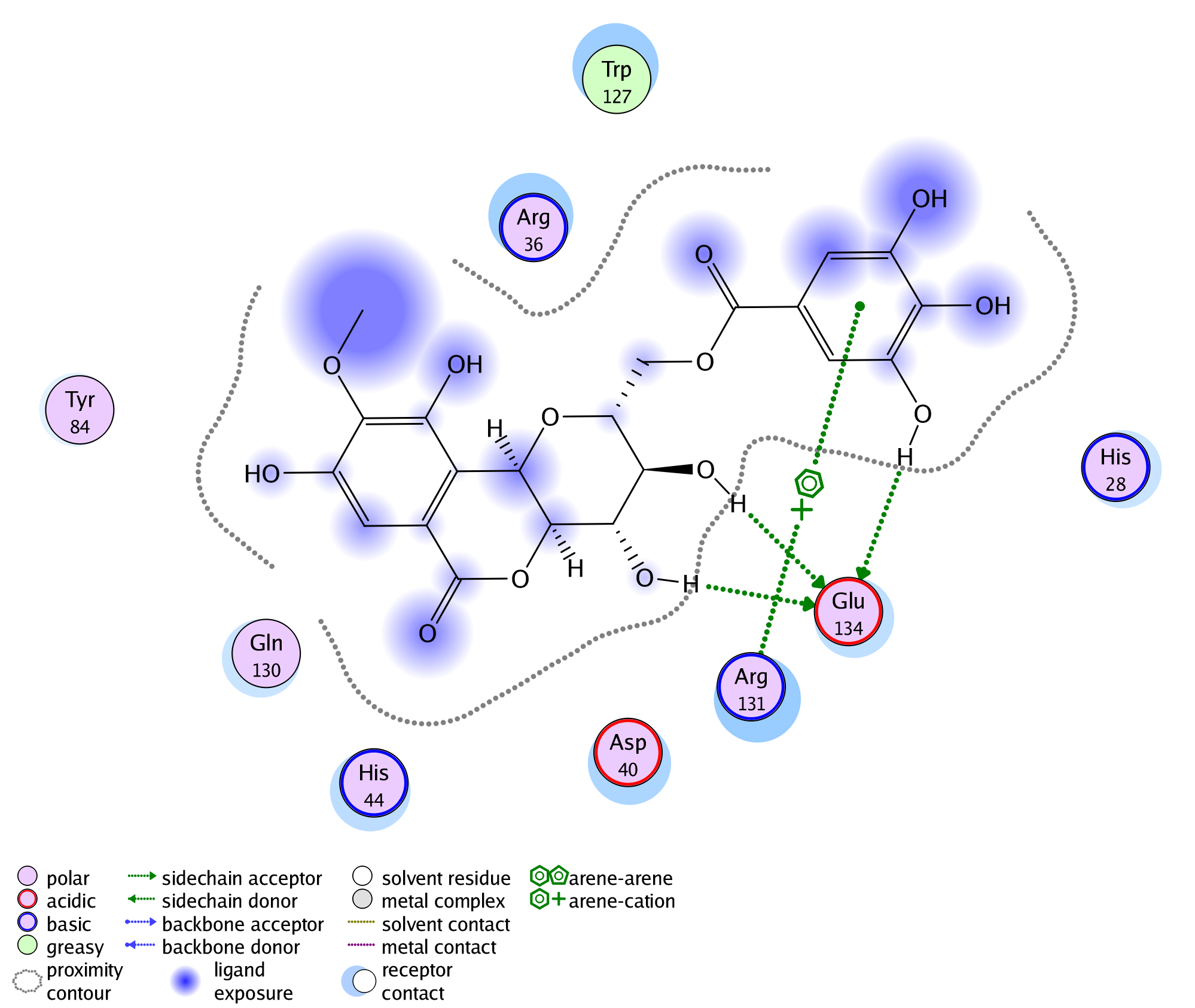


FIGURE S6: 2D ligand interaction diagram of the docked ligand (compound **2**) within the binding site of the protein of *Plasmodium* PDB code PFG27.


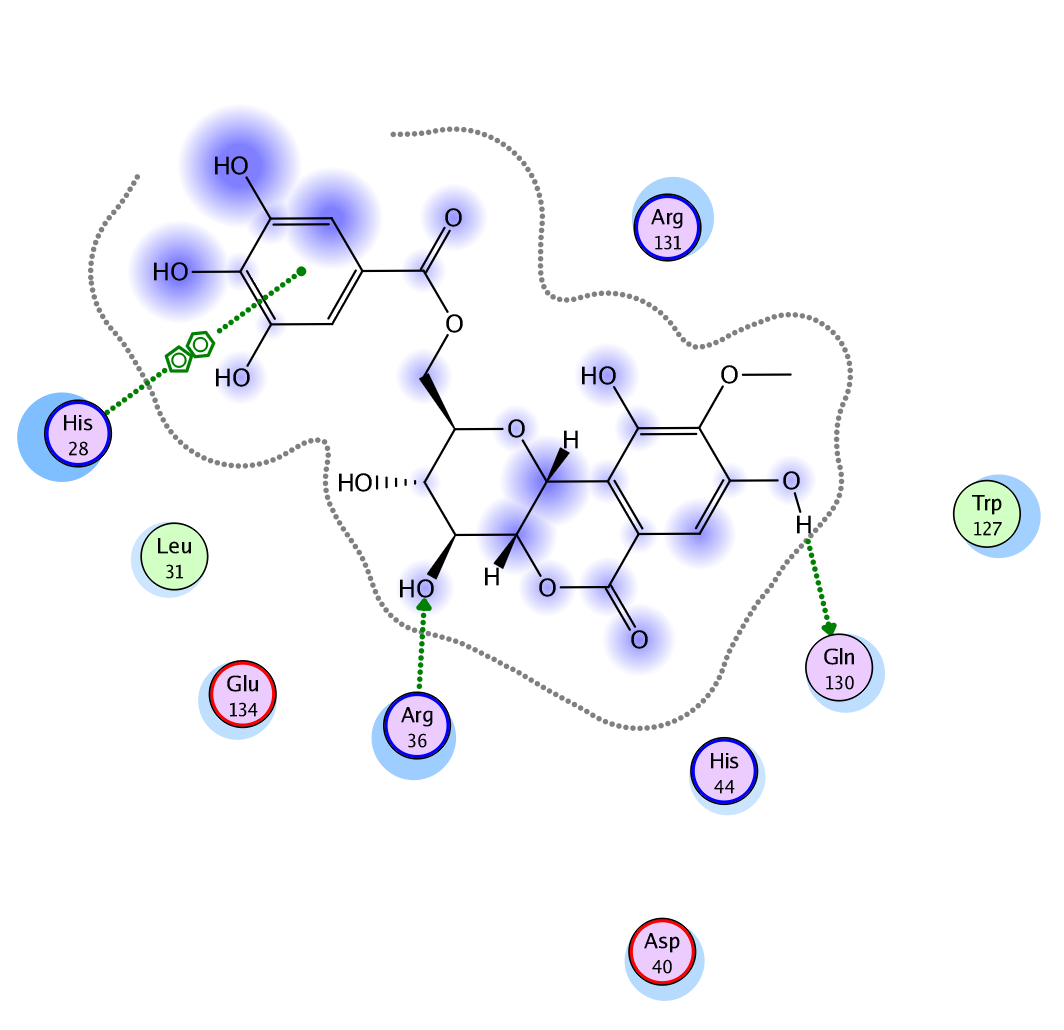


FIGURE S7: 2D ligand interaction diagram of the docked ligand (compound **2**) within the binding site of the protein of *Plasmodium* PDB code PFG27.


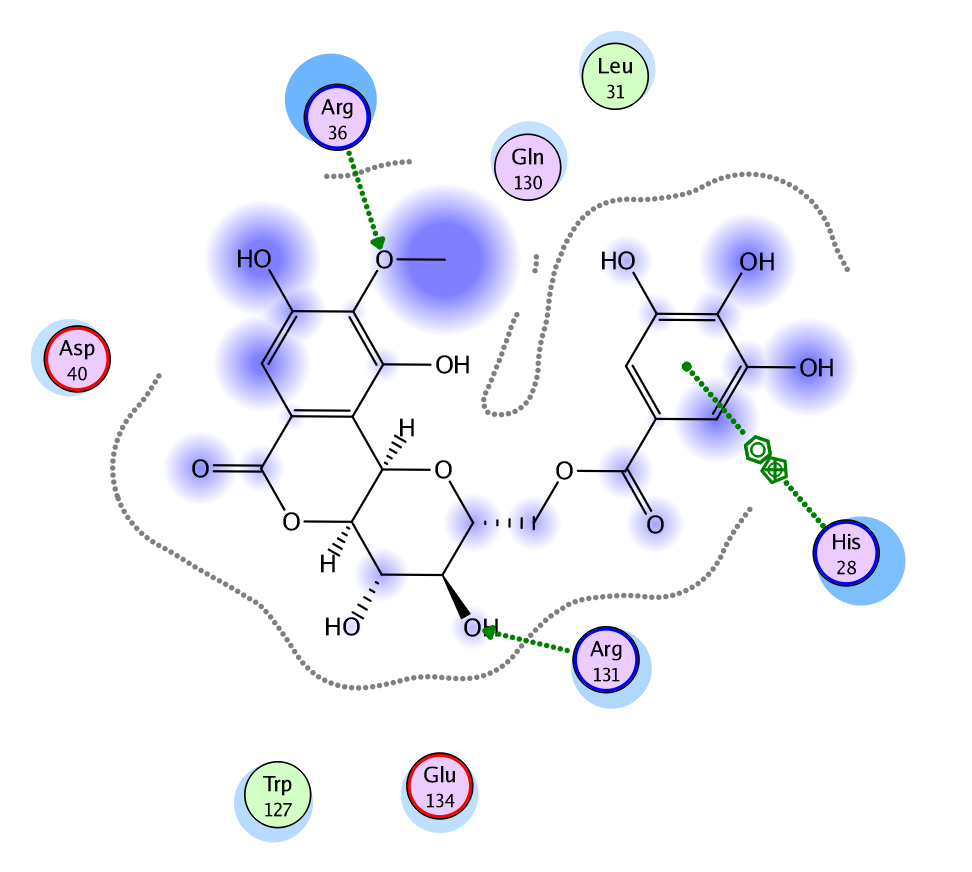


FIGURE S8: 2D ligand interaction diagram of the docked ligand (compound **2**) within the binding site of the protein of *Plasmodium* PDB code PFG27. The Figure is rendered by MOE.
